# Process evaluation for OptiBIRTH, a randomised controlled trial of a complex intervention designed to increase rates of vaginal birth after caesarean section

**DOI:** 10.1186/s13063-017-2401-x

**Published:** 2018-01-05

**Authors:** Patricia Healy, Valerie Smith, Gerard Savage, Mike Clarke, Declan Devane, Mechthild M. Gross, Sandra Morano, Deirdre Daly, Susanne Grylka-Baeschlin, Jane Nicoletti, Marlene Sinclair, Rebekah Maguire, Margaret Carroll, Cecily Begley

**Affiliations:** 1 School of Nursing and Midwifery, National University of Ireland, Galway, Ireland; 2 School of Nursing and Midwifery, Trinity College Dublin, Dublin, Ireland; 3 Centre for Public Health, Institute for Health Sciences, Queen’s University Belfast, Belfast, Northern Ireland; 4 Midwifery Research and Education Unit, Hannover Medical School, Hannover, Germany; 5 Department of Neurologic, Oculist, Gynaecologic, Maternal and Infant Sciences, University of Genoa, Genoa, Italy; 6 Centre for Maternal, Fetal and Infant Research, Institute of Nursing and Health Research, School of Nursing, Ulster University, Antrim, Northern Ireland; 7 Institute of Health and Care Sciences, Sahlgrenska Academy, University of Gothenburg, Gothenburg, Sweden

**Keywords:** Process evaluation, Fidelity, Complex intervention, Randomised controlled trial, VBAC, Midwifery, Ethnography

## Abstract

**Background:**

Complex interventions encompassing several interconnecting and interacting components can be challenging to evaluate. Examining the underlying trial processes while an intervention is being tested can assist in explaining why an intervention was effective (or not). This paper describes a process evaluation of a pan-European cluster randomised controlled trial, OptiBIRTH (undertaken in Ireland, Italy and Germany), that successfully used both quantitative and qualitative methods to enhance understanding of the underlying trial mechanisms and their effect on the trial outcome.

**Methods:**

We carried out a mixed methods process evaluation. Quantitative and qualitative data were collected from observation of the implementation of the intervention in practice to determine whether it was delivered according to the original protocol. Data were examined to assess the delivery of the various components of the intervention and the receipt of the intervention by key stakeholders (pregnant women, midwives, obstetricians). Using ethnography, an exploration of perceived experiences from a range of recipients was conducted to understand the perspective of both those delivering and those receiving the intervention.

**Results:**

Engagement by stakeholders with the different components of the intervention varied from minimal intensity of women’s engagement with antenatal classes, to moderate intensity of engagement with online resources, to high intensity of clinicians’ exposure to the education sessions provided. The ethnography determined that, although the overall culture in the intervention site did not change, smaller, more individual cultural changes were observed. The fidelity of the delivery of the intervention scored average quality marks of 80% and above on repeat assessments.

**Conclusion:**

Nesting a process evaluation within the trial enabled the observation of the mode of action of the intervention in its practice context and ensured that the intervention was delivered with a good level of consistency. Implementation problems were identified as they arose and were addressed accordingly. When dealing with a complex intervention, collecting and analysing both quantitative and qualitative data, as we did, can greatly enhance the process evaluation.

**Trial registration:**

Current Controlled Trials Register, ISRCTN10612254. Registered on 3 April 2013.

**Electronic supplementary material:**

The online version of this article (doi:10.1186/s13063-017-2401-x) contains supplementary material, which is available to authorized users.

## Background

The interventions being tested in health services research are becoming increasingly more complex, encompassing several interconnecting and interacting components [1]. Although the primary outcome is the main focus of complex research studies, consideration of the study processes, including design and execution, is equally important. Evaluation of the underlying processes of a complex study assists in explaining why an intervention has failed unexpectedly or has unforeseen consequences or why a particular intervention is successful in achieving its outcome, and how that success can be optimised [1]. Information about the process of a study is also very helpful for study replication.

Process evaluations are particularly important in cluster randomised trials, where the studies take place across several sites with a variety of contexts in which the intervention is both delivered and received. In these situations a ‘standardised’ intervention may, in reality, be implemented in different ways or be the subject of different reactions [2]. A resulting ‘lack of effect’ may thus merely indicate problems with implementation rather than true ineffectiveness [3]. Variation in intervention delivery has implications for both internal and external validity and reduces the potential for generalisability of findings.

A process evaluation used within a trial is a useful way of assessing how well the trial protocol was adhered to [3]. This can help explain how well all aspects of the protocol were followed with regard to recruitment, treatment fidelity, data collection and reporting. This structured process can help identify issues and factors that may have caused a deviation in expected outcomes, and it may illuminate local contextual factors which could have impacted the trial implementation, which might not otherwise have become apparent to the trial team.

The OptiBIRTH trial (ISRCTN10612254) research team developed a process evaluation plan to explore and document the implementation of the various aspects of the complex OptiBIRTH intervention and how it was received by key stakeholders, as well as to identify factors that could explain variation in outcomes, if any, across intervention sites. In this paper, we report on the OptiBIRTH process evaluation procedures, fidelity to the intervention at the 15 trial sites and preliminary findings from the embedded ethnographic study. The OptiBIRTH trial results will be reported separately. The Template for Intervention Description and Replication (TIDieR) checklist was used to guide the structure and content of this paper [4].

### Context

The OptiBIRTH trial was a cluster randomised controlled trial, with randomisation at the maternity unit level, in three European countries with relatively low vaginal birth after caesarean section (VBAC) rates. The trial took place across 15 small (<2000 annual births), medium (2000–5000 annual births) and large maternity units (5000–8500 annual births) in both urban and rural locations in Ireland, Italy and Germany, with baseline VBAC rates in 2012 of 34% in Germany, 32% in Ireland and 8% in Italy. In all three countries, publicly funded maternity care is provided free of charge, but there is also a private healthcare model existing in parallel. There are some variations between countries in the way services are delivered, but the units in the OptiBIRTH trial provided a model of care that was obstetrics-led, and all births occurred in hospital. In general, women book at the maternity hospitals for antenatal care at around 19–20 weeks of gestation in Ireland, 24–26 weeks in Italy and 28–32 weeks in Germany (although these women attend primary care centres for their initial maternity care). Multiparous women in all three countries would usually be in the range of 33–36 years of age, and 10–20% would have three or more children. National guidelines on VBAC existed prior to the OptiBIRTH trial in Ireland, and guidelines on VBAC were included in a guideline on caesarean section (CS) in Italy [5, 6]. A national VBAC guideline was withdrawn in Germany because it was considered to be outdated. The objective of the trial, as set out in the published protocol, was to compare the effectiveness of usual care versus an intervention that had been developed with the intention of maximising VBAC [7]. A total of 2002 women were recruited to participate in the trial across the 15 sites in the 3 countries (1195 in the intervention arm and 807 in the control arm). Because the intervention was complex with multiple interconnected components that were delivered to a variety of stakeholders (pregnant women, midwives, obstetricians) at different times, we planned a parallel process evaluation to assess adherence to agreed intervention delivery content and methods and to enhance understanding of the underlying trial mechanisms. The objectives of the process evaluation, designed on the basis of existing theoretical and methodological literature, were as follows:

- To provide information on the intervention and how it was planned to be delivered [8, 9]
- To provide information on the context in which the intervention was implemented [8, 9]
- To measure the interaction between the intervention and the context [8] (i.e., the response of clusters and individuals to the intervention [10])
- To ascertain the fidelity of the intervention and how closely it matched the intended intervention [10]
- To explore the mechanisms of adaptation and change [11] occurring in one Irish site as the intervention was introduced

### Information on the intervention

The OptiBIRTH intervention was developed on the basis of evidence from three systematic reviews on interventions designed to increase VBAC rates [12–14] and from findings from focus group interviews with 115 clinicians [15, 16] and 71 women [17, 18] in 6 countries, 3 with high (Finland, the Netherlands, Sweden) and 3 with low (Ireland, Italy and Germany) VBAC rates. Through an iterative process with key stakeholders and using motivational theory to guide its formation, the final complex intervention consisted of multiple interconnected components (Table 1).

**Table 1:** Interconnected components of the intervention

Intervention components
Appointment of local clinical opinion leaders in midwifery and obstetrics
Motivationally enhanced educational materials for women, which were conveyed in two face-to-face education sessions
Information for clinicians on VBAC and repeat caesarean section provided in a 1-h information session
An OptiBIRTH website subdivided into two sections: one for women called ‘icanbirth’ and one for clinicians called ‘shecanbirth’

*VBAC* Vaginal birth after caesarean section

Once the clinical leaders of a study site had agreed to the site’s participation in the trial, all clinicians were included as agents of intervention conduct. Clinical opinion leaders (OLs) in midwifery (MOL) and obstetrics (OOL) from the site staff were appointed by interview. The MOLs’ role was to promote and support VBAC, to act as research assistants for the study and to deliver the intervention. The OOLs’ role was to promote and support VBAC, to deliver the intervention and to support the MOLs. A 1-day training programme for OLs was developed by the core research team, consisting of the co-ordinator, project manager, country principal investigators (PIs) and country post-doctoral researchers [19]. The training was delivered by the national OptiBIRTH research team (PIs and post-doctoral researchers in each country) to all OLs in the nine intervention sites (three per country). A follow-up meeting, attended by all OLs, the PI and the post-doctoral researcher in each country, took place at the end of the pilot study or in the first few weeks of the main study to encourage sharing of successful techniques for recruitment and ideas for improvement in implementation. The OLs attended online and face-to-face training sessions on how to access and use the materials on the website. The OLs subsequently provided the access information to women and clinicians as part of the intervention. They also each received a comprehensive manual detailing a precise, structured programme of how exactly the clinicians’ information sessions and women’s education sessions were to be delivered.

Aspects of the OptiBIRTH intervention were designed to be delivered to the maternity staff in the maternity unit clusters. Cluster-level aspects of the intervention included information for clinicians on VBAC and rising CS rates, the benefits and risks related to VBAC and repeat CS, and ways to increase VBAC rates, all provided in a 1-h information session. Delivery of the clinician’s information sessions commenced as soon as the recruitment of women began in the antenatal clinics and continued until the last OptiBIRTH baby was born. The information and all relevant published papers referred to in those sessions were available in a secure section of the OptiBIRTH website, called ‘shecanbirth’, to which clinicians were given a site-specific password. All clinicians providing care to pregnant women were expected to attend this session at least once during the study. The intention was that cluster-level delivery of the intervention would influence the underlying culture of the maternity unit where pregnant women received their care.

Further aspects of the OptiBIRTH intervention were designed to be delivered to the individual pregnant women attending the maternity units. These included motivationally enhanced educational materials for women, which were conveyed in two face-to-face education sessions of 2 h each. Women were able to bring significant others (partners, mothers, children) with them to these sessions if they desired. The first session, facilitated at 24–31 weeks of gestation, addressed the previous CS birth and introduced the topic of VBAC. The second session, facilitated at 31–35 weeks of gestation, was focused on preparing for birth in the current pregnancy. Delivery of the antenatal classes for women commenced as soon as the first recruited woman reached 24 weeks of gestation and continued until the last OptiBIRTH baby was born. However, because these women were not first-time mothers, we anticipated that they would have challenges regarding attendance at antenatal classes, so the PowerPoint presentations used for the classes, with an accompanying voice-over, were also provided on the study website to enable a greater proportion of women to engage with them. The online component (consisting of the PowerPoint presentations shown in the face-to-face antenatal classes and three phone applications [‘apps’]), including a birth planner, was available for women in a secure section on the OptiBIRTH website, called ‘icanbirth’, to which they were given an individual password. The online resources were available in English, German and Italian. Women could seek individual support from the MOLs by telephone, face-to-face meeting, or through the website (up to a total of 2 h per week). The intention was that delivery of the intervention at the individual woman level would influence the extent to which women felt informed and engaged in decision-making about their choice of mode of birth and thus could and would achieve their planned VBAC.

Women in the intervention sites could participate at two levels: consent to receive the full OptiBIRTH intervention (i.e., plan to attend the antenatal classes) or consent to provide only ‘routine data’ (i.e., chose not to attend/engage with the classes and the online resources but give the research team permission to access their healthcare records). The aim of this process evaluation was to monitor the implementation of the OptiBIRTH intervention throughout the trial, collect data on aspects of the process of intervention implementation and assess fidelity to the intervention so that the relationship between trial outcomes and differences in the amount and quality of the intervention at each site could be explored.

## Methods

We used a combination of both qualitative and quantitative methods to monitor the fidelity of the intervention, the response of the clusters and the response of pregnant women to the intervention as well as the mechanisms of adaptation and change at the intervention site as described in the subsections below.

### The fidelity of the intervention

*Intervention fidelity* refers to the degree to which the prescribed components of the intervention, as described in the study protocol, have been delivered [20]. The extent to which the OptiBIRTH intervention was delivered as intended and the quality of that delivery were assessed during formal fidelity checks designed by the research team. A post-doctoral researcher observed the OLs’ delivery of the education sessions to women and the delivery of the 1-h information session to clinicians to check adherence to the planned, structured delivery. In each trial country, the same researcher visited all three intervention sites on two occasions at least 6 weeks apart, once in the pilot study phase and once in the early main study phase, to observe and record fidelity to the agreed delivery methods. We developed a structured observational checklist to measure fidelity while we observed the OLs delivering the sessions. The checklist criteria were based on the standardised training and the OL manual we had designed. The checklist of pre-determined criteria was administered consistently by a member of the research team not involved in delivering the intervention. The checklist for both the women’s and the clinicians’ sessions consisted of items related to the degree of adherence by the facilitator to the education classes’ planned content and the quality of the delivery of the intervention components, rated either dichotomously (yes, done as planned; or no, not as planned) or using a Likert scale. Nonspecific factors such as empathy, communication style, credibility, engagement and sensitivity were rated using a Likert scale. The tool comprised 111 items for monitoring the women’s class and 86 items for the clinicians’ class, from which we calculated the overall score for the quality mark. We created an a priori specification of the ideal and minimally acceptable quality mark for delivery of the women’s classes and the clinician’s information sessions (Table 2).

**Table 2:** Rating scale for the quality of delivery of the classes and information sessions

High quality	Both antenatal classes delivered with an average quality mark of at least 80%
Moderate quality	Both antenatal classes delivered with an average quality mark of 65–79%
Low quality	Both antenatal classes delivered with an average quality mark of 50–64%

### The response of pregnant women

This refers to the extent to which the individual recruited women engaged with the various components of the OptiBIRTH intervention. We created an a priori specification of the ideal and minimally acceptable treatment dose (Table 3). The *treatment dose* refers to the amount of the components of the intervention delivered by implementers and the extent to which participants received and used materials or other resources [19, 20]. This provides information about the degree to which the intended audience engaged with the intervention. In our case we measured treatment dose by documenting attendance by the women at the antenatal classes.

**Table 3:** Rating scale for the intensity of the intervention (women)

High intensity	>60% of recruited women attending two antenatal classes	or	>80% of recruited women attending one antenatal class
Moderate intensity	>50% of recruited women attending two antenatal classes	or	>65% of recruited women attending one antenatal class
Low intensity	>40% of recruited women attending two antenatal classes	or	>50% of recruited women attending one antenatal class
“No intervention”	0–40% of recruited women attending two antenatal classes	or	0–50% of recruited women attending one antenatal class

We also monitored the online activity to assess levels of engagement by the OptiBIRTH women with the online components of the intervention. A survey was created using the QUALTRICS web-based tool (www.qualtrics.com) for completion by those using ‘icanbirth’, which was the secure section on the OptiBIRTH website for women. To monitor the amount of individual support women sought, the MOLs logged the number of individual telephone or face-to-face contacts from women recruited into the study and documented the total time spent advising women.

### The response of the clusters

This refers to the extent to which the maternity units and professionals therein adopted (or not) the intervention into their existing systems and everyday work [10]. The results of the fidelity checks and documentary evidence of attendance rates at the clinicians’ information sessions informed this section. Again, we created an a priori specification of the ideal and minimally acceptable treatment dose (Table ***4***), which in this case was clinicians’ class attendance rates.

**Table 4:** Rating scale for the intensity of the intervention (clinicians)

High intensity	>70% of clinicians attending the information session
Moderate intensity	>60% of clinicians attending the information session
Low intensity	>50% of clinicians attending the information session
“No intervention”	0–50% of clinicians the information session

We also monitored online activity to assess levels of engagement by the clinicians with the online components of the intervention. A survey was created on QUALTRICS for completion by those using ‘shecanbirth’, which was the secure section on the OptiBIRTH website for clinicians.

### The mechanisms of adaptation and change in the intervention site

An ethnographic study was conducted at one of the intervention sites in Ireland to explore the mechanisms of adaptation and change following the introduction of the intervention [21]. A 16-month period of observation took place throughout the study. Twelve clinicians and 15 women were interviewed before, during and after the introduction of the intervention. The MOL at the site was interviewed three times and the OOL once throughout the study period.

## Results

### The fidelity of the intervention

Fidelity checking during delivery of the intervention yielded average quality marks of 80% and above at all sites. During fidelity checking and other random site visits by the post-doctoral researcher, OLs were observed to be delivering the intervention consistently and to a level of high quality.

### The response of pregnant women to the intervention

There were 1073 women recruited to receive the full OptiBIRTH intervention. A further 122 women chose the ‘routine data only’ participation, which gave us permission to access the healthcare records for the woman and her baby, but those women chose not to engage with the classes and the online resources [7]. The documentary evidence of attendance rates at the women’s education sessions showed that in Ireland, 42% (162 women) of OptiBIRTH women attended class 1 and 37% (143 women) attended class 2; in Germany, 19% (91 women) attended class 1 and 17% (81 women) attended class 2; and in Italy, 54% (166 women) attended class 1 and 38% (117 women) attended class 2. These rates were low and correspond to an a priori level of ‘no intervention’. Women rated the usefulness of these classes on a 10-point Likert scale, with 0 being of no use at all and 10 being the most useful, in a 3-month postnatal survey. Ninety percent of the 341 women who attended both classes rated the classes in the top 5 points of the scale, with 40% scoring the classes as 9 or 10 on the scale.

**Fig. 1:**
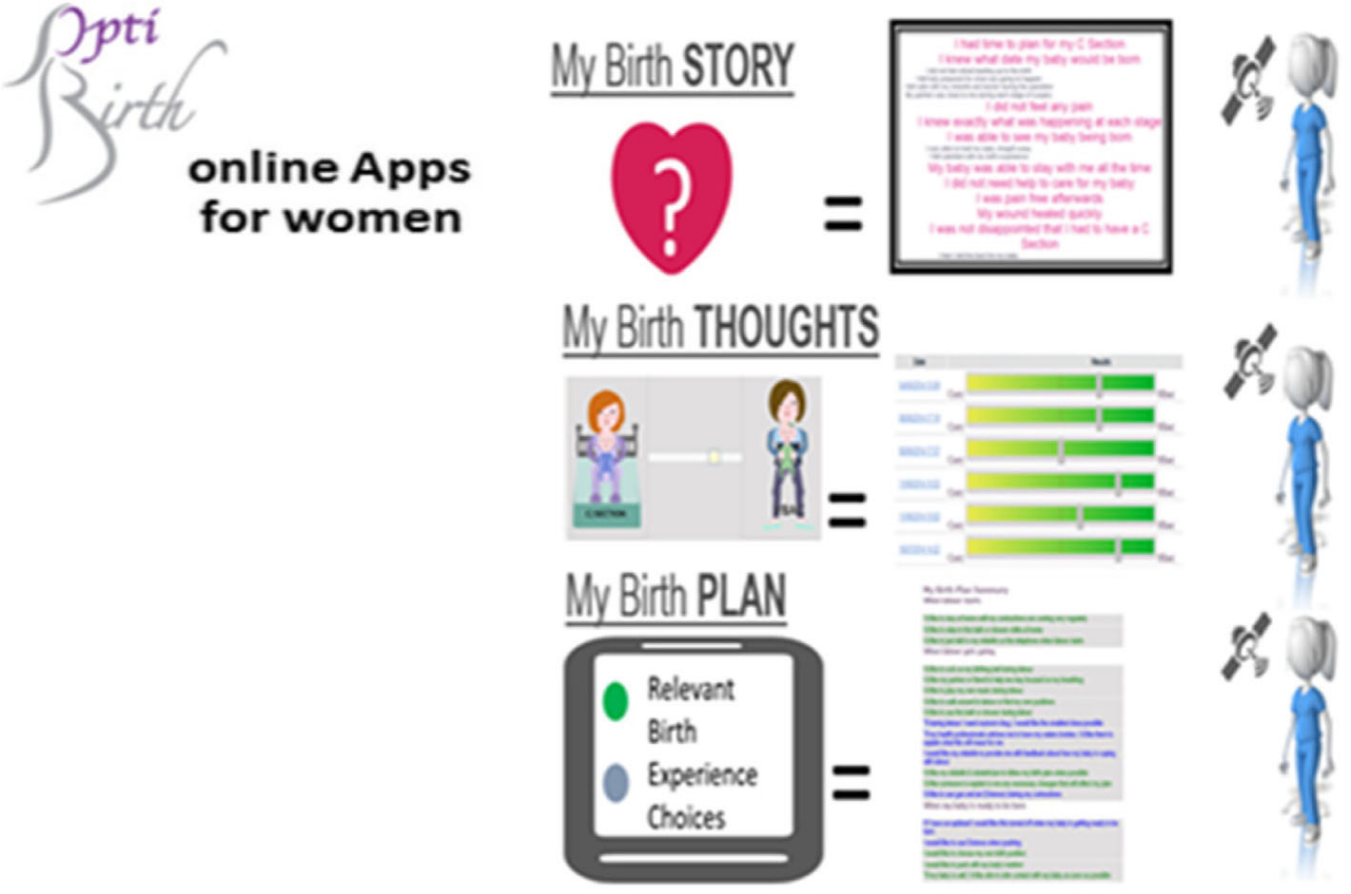
Online apps designed for the OptiBIRTH intervention

An evaluation of the online environment and the apps was an integral component of the overall project. The frequency of log-ins, including repeat and unique visitors, to the OptiBIRTH website was documented. There were 2608 visits by women recorded on the OptiBIRTH website. Among the women engaging with the OptiBIRTH intervention, for the trial as a whole, 97% (*n* = 1037) of the 1073 women recruited to receive the intervention registered to use the online resources. Of those who registered, 55% (*n* = 570) logged in to the website a total of 2608 times. Three online apps were designed to be completed sequentially: app 1 had to be completed before app 2, and app 2 had to be completed before app 3 (Fig. 1). Our analysis shows that 30% of registered women (*n* = 173) completed app 1, 46% (*n* = 79) of these women completed app 2 and 57% (*n* = 45) completed app 3. The overall app use for women recruited to receive the intervention (*n* = 1073) was 16% for app 1, 7% for app 2 and 4% for app 3 Additonal file 1.

Logs of the individual support provided by the MOLs to women revealed that although many women did not request any more support than that provided by the classes and the online components, a small number of women required intense personal support. In addition, the MOLs logged a significant amount of time spent on individual contacts around consent reminders and class attendance arrangements. Over the duration of the study, the individual contact provided by the MOLs to women averaged more than the 2 h per week that had been anticipated.

### The response of the clusters

Attendance lists at the clinicians’ sessions were maintained, duplicates were removed (many clinicians attended more than once) and proportions were calculated using whole time equivalent numbers provided by human resources departments. In Ireland 80% of midwives and 80% of doctors attended. In Germany attendance rates were 80% of midwives and 95% of doctors, whereas in Italy 52% of midwives and 57% of doctors attended. This corresponds to a high intensity achieved for the clinicians’ interventions in Ireland and Germany. Italy achieved a lower level of intensity. The frequency of log-ins, including repeat and unique visitors, to the OptiBIRTH website was documented. There were 443 visits by clinicians recorded on the OptiBIRTH website.

### The mechanisms of adaptation and change in one Irish site

The observational, field notes and interview data collected during the ethnographic study conducted at one of the intervention sites in Ireland showed that the intervention underwent a process of ‘ritualisation’ to become embedded and accepted as ‘routine’ in daily practice. Women also developed study-specific identities; that is, they became ‘OptiBIRTH women’ by virtue of participating in the study. Transference of authoritative knowledge, in particular, during the educational classes was also observed as a central tenet of intervention delivery. Lastly, the effect of various power positions (women-clinicians, women-women, midwives-obstetricians) also affected intervention delivery, including how it was viewed, accepted and implemented at the field site. The ethnography demonstrated that although the overall culture in this intervention site did not change, smaller, more individual cultural changes were observed, in particular observations of women emerging as the main drivers of change around VBAC as the intervention phase of the study progressed [22].

## Discussion

The design and execution of research studies become more challenging as the interventions being evaluated move from simple towards more complex interventions with several components [3]. This is further compounded when such studies take place across several sites in several countries with multiple distinct work packages within the study design, as was the case with the OptiBIRTH trial. The OptiBIRTH trial took place in 15 maternity units across 3 different countries. Birth rates in the three countries differed, with 1.95 per woman in Ireland, 1.37 in Italy and 1.47 in Germany [23]. Understanding of the outcomes of such complex trials, conducted in a variety of practice settings, could be greatly improved by better understanding of the processes of the trials. Smyth et al. suggested that accounts of the reality of undertaking research, with all its challenges, are missing from the currently available literature [24]. Process evaluations nested within trials may address this deficit. It is widely recognised that such process evaluations move beyond exploring whether an intervention works but also explore how the intervention works, why it works (or not), when it works, who is needed to make it work, what is needed to make it work, and if it can work again in other contexts, including barriers to its being adopted and having effect.

Conducting a process evaluation parallel to the OptiBIRTH trial enabled us to ensure that the intervention was delivered appropriately. Fidelity monitoring identified implementation problems as they arose and allowed us to address them accordingly. Observations by a post-doctoral researcher as a non-participant of the OLs delivering the education and information sessions removed the potential for social desirability bias that may have occurred with self-reported assessment by the OLs. The initial observations at the beginning of the trial revealed minor issues around confidence with delivery and command of the subject matter. An action plan was implemented in which the post-doctoral researcher explained to the OLs the evidence informing the intervention and role-played the delivery of that evidence in the sessions. The MOLs were very comfortable and confident engaging with the OptiBIRTH women, but less so about presenting to their peers, especially their obstetrics colleagues whom they felt would ask them questions they were unable to answer. The availability of the post-doctoral researcher and the obstetric OL to accompany the MOLs in a coaching capacity at the beginning of the trial when they were delivering the sessions was instrumental in building their confidence and their technique. During the clinicians’ sessions, difficulty with delivery at an appropriate pace was identified and was due to an excess of material to be delivered within the hour-long clinicians’ session. This was addressed by re-organising and/or removing some of the content. The difficulties resolved as the OLs became more confident and more familiar with the literature informing the sessions. Average quality marks of 80% and above were achieved on repeat assessments in all sites. Although there is a lack of clear guidelines about what the optimal level of adherence should be, most agree that 80–100% constitutes high fidelity, whereas 50% constitutes low fidelity [19]. In addition to acting as a support for the OLs, regular site visits by the post-doctoral researcher enabled quality assurance throughout the trial. For the OptiBIRTH study, a post-doctoral researcher acted in this capacity, but for other studies this role could be fulfilled by a project manager or a research supervisor.

Balancing fidelity in terms of ‘adherence to intervention’ versus application to context and flexibility to respond to individuals’ needs was challenging. Context-level adaptation does not necessarily mean that the integrity of what is being delivered and evaluated across multiple sites is lost, but uncontrolled variations in the implementation of the intervention may threaten the study’s integrity [25, 26]. The context within which intervention studies take place may sometimes challenge the way in which interventions are delivered [27], and the variety of practice settings was a particular complexity in this trial. We needed to preserve the core components of the intervention but facilitate the flexibility required by the practice settings. The research team decided that it was preferable to have a structured programme requiring strict adherence to a set format because the trial covered three countries, with three intervention sites in each, and had to be designed to be suitable for transfer to all European countries. We standardised the training for the OLs delivering the intervention to ensure that the intervention was delivered systematically and consistently. A structured training manual offered the advantages of greater consistency and precision in the delivery of the intervention and enhanced internal validity [28]. Thus the OptiBIRTH training manual and the observation tool for assessing fidelity were very structured around the core components of the intervention. However, flexibility was accommodated around secondary aspects such as the timing and location of the women’s classes. The clinicians’ session was designed in such a way that it could be delivered as a single 1-h session or split into four 15-minute sessions as the need arose. This enabled the delivery of the intervention to be maximised while being responsive to local contexts and allowed us to deliver and evaluate a very structured intervention in a ‘real-world’ practice context. We believe that the highly structured manual used in the context of very experienced OLs delivering the intervention allowed us to achieve the correct balance between standardisation for internal validity and flexibility for external validity.

We were able to observe the mode of action of the intervention in its practice context by monitoring the response of the maternity unit cluster and the pregnant women to the intervention. Our process evaluation revealed that there was a variable level of engagement by stakeholders with the different components of the intervention. This varied from minimal intensity of women’s engagement with antenatal classes, to moderate intensity of engagement with online resources, to high intensity of clinicians’ exposure to the education sessions provided.

Preferential recruitment was not the method used for this trial; however, women could choose whether they took part in two ways, either as full participants or as ‘routine data only’ participants, and those who agreed to join and to attend classes may have been more interested in achieving a VBAC. This will have had no effect on the trial outcome, because the primary outcome was the VBAC rate after trial completion compared with the VBAC rate in the year prior to the trial for all women at the study sites.

The low intensity of women’s engagement with antenatal classes, though disappointing, was not altogether surprising. Engaging women who are not first-time mothers with antenatal education is notoriously difficult. A national survey of women’s experience of maternity care conducted in the United Kingdom in 2010 found that only 12% of multiparous women compared with 67% of first-time mothers attended antenatal education [29]. The OptiBIRTH women already had children, so child-minding while they attended classes may have been a challenge. The classes were delivered between 24 and 35 weeks of gestation and so occurred before maternity leave had begun. The OLs were very pragmatic and adaptable in trying to mitigate those challenges, and they tried to deliver the classes at a variety of times, including morning, midday, evenings and even weekends, to accommodate the women. They ensured that the infrastructure in which they delivered the sessions was safe and suitable for any woman wanting to bring children with her. Classes were provided for any number of women who were able to attend, regardless of whether it was as low as 2 women or as many as 15 women. However, despite all their efforts, attendance at the classes was less than we would have liked. The women who did manage to attend the classes rated them very highly in their follow-up 3-month postnatal survey.

Perhaps some of the solution to poor attendance at antenatal education classes lies in the online resources. There were 3051 visits by users (2608 by women, 443 by clinicians) recorded on the OptiBIRTH website, suggesting that there is potential for the website to be improved and developed further to provide a more interactive environment where women could attend a virtual class. However, further research is required to establish women’s preferences. The OptiBIRTH women attending the classes seemed to really appreciate the opportunity to not only engage freely with an obstetrician and midwife but also with other women who had experienced a previous CS and were now negotiating a birth after a previous CS. The ethnographic findings around the women forming a new identity as ‘OptiBIRTH women’ suggests a collective identity emerging from meeting other women at the classes, something that may not happen in an online forum.

A high intensity of clinicians’ exposure to the education sessions provided was achieved in Ireland and Germany. The information sessions for all clinicians at each site were delivered by the OLs on a rolling basis so that each clinician would attend the session at some time during the trial. Again, the OLs were very pragmatic and adaptable in trying to mitigate challenges around attendance at these sessions. The information to be delivered required 1 h in total but was formatted in such a way that it could be delivered in four distinct 15-minute sessions if required. An example of the practical application of this flexibility occurred where one MOL delivered the 15-minute sessions on 4 successive nights to her midwifery colleagues who were on a week of night duty. The 15-minute sessions also worked well in areas such as the antenatal clinics where staff worked stable hours as opposed to shifts. Rather than ask busy clinicians to find another hour in their already packed schedules, the OLs tried to deliver the sessions wherever possible within already existing meetings such as weekly risk management meetings, perinatal case study meetings, midwifery case review meetings or further education classes. The OptiBIRTH clinicians’ session became part of the mandatory induction week for newly appointed junior doctors in many of the sites. From recruitment of the first OptiBIRTH participant to the birth of the last OptiBIRTH baby, the study lasted 21 months and so spanned a number of rotations of junior doctors and new midwifery staff. Being able to meet those doctors and midwives at induction ensured that they were aware of the study and that they also received evidence-based information about VBAC as soon as they began working at the study site. The senior obstetricians and midwives were instrumental in ensuring that their teams of junior clinicians attended the sessions but also role-modelled the behaviour by attending the sessions themselves. Having senior level buy-in is vital for all change processes. To encourage attendance at the sessions, the MOLs at each site collected data on study eligibility rates, recruitment rates, VBAC, and elective and emergency repeat CS rates, and they presented these data to clinicians quarterly at the end of the session. The clinicians’ exposure to the education sessions ensured that clinicians gained evidence-based knowledge on VBAC which helped VBAC become a legitimate option for women with a previous CS and helped clinicians and women jointly make an informed decision about trying for a VBAC.

The lower level of engagement by clinicians in Italy is most likely due to a culture that is less receptive to the concept of VBAC. There are a number of reasons for this, among them being the highly medicalised models of care for pregnancy and childbirth delivered largely by obstetricians in a private setting with midwives having a minimal role, limited skills among those clinicians around VBAC, and a widespread culture of fear and risk that leads to a specialists bias in dealing with CS and VBAC. The pre-trial VBAC rates in Italy were extremely low at only 8%.

Although it was not possible to measure the nature of the change in the attitudes or behaviours of the healthcare staff providing care to the pregnant women directly, it is possible to measure changes in the rates of VBAC in the sites. Descriptions of the contexts in which interventions are implemented may help improve understanding of the intervention’s effectiveness, the findings of the study and their generalisability [3, 25]. In addition to the contextual complexity of multiple countries and multiple sites, the OptiBIRTH intervention was delivered at sites that had low but variable background VBAC rates at the beginning of the trial. The baseline rate of VBAC for women with one previous CS for a 12-month calendar year preceding the start of the study was recorded at each site prior to introduction of the intervention. That information gave an indication of the attitude towards VBAC from both women and clinicians at that site. Such attitudes at both individual and cluster levels may influence response to the intervention. VBAC baseline rates for the year after the completion of the study (2016) have been collated and are currently being analysed. This will provide an indication of the degree of change in the care that clinicians delivered to the women they encountered and will also provide some information about sustainability (or not) of the effect. The embedded ethnographic study seems to be suggesting that the mechanism of adaptation and change, although slower than expected, was beginning to effect change towards the end of the trial period [21]. The embedded ethnographic study was conducted at one intervention site but has potential transferability to other intervention sites.

The combination of both quantitative and qualitative methods, including checklists, researcher observations, and interviews, to monitor intervention implementation at the OL (deliverer), individual woman (recipient) and maternity unit staff (cluster) levels will make a valuable contribution to explaining the outcomes of the study and enhancing the credibility of the evidence. It also provides a data trail of what happened during the implementation of the intervention. Embedding an ethnographic study at one site enabled us to explore midwives’, obstetricians’ and women’s experiences of the intervention and their responses to it. This has significant implications for dissemination of the study findings but also for replication of the study in the future.

### Limitations

Craig et al. acknowledged that there is no single best way to conduct a process evaluation and suggested that process evaluations need to be tailored to the trial, the intervention and the outcomes being studied [3]. For pragmatic and resource reasons, we had to decide where to focus our attention for the purpose of the process evaluation for OptiBIRTH. There were certain aspects of the process on which we were able to gather data (e.g., numbers attending both the women’s and clinicians’ classes), but other aspects for which we were less able to gather data (e.g., behavioural change in practitioners), which would have required a discrete substudy. When implementing a complex intervention, using a combination of quantitative and qualitative data as we did can greatly enhance the process evaluation. The processes involved in the trial and intervention implementation, described above, were funded as part of the trial and supported by a research team. Such support necessarily ceases once funding ceases, and often initiatives founder at that stage. Further important questions are thus how, and why, these processes are sustained over time [8]. In the OptiBIRTH trial, hospitals agreeing to join the study also committed to continuing the intervention after trial end if the intervention was found to be beneficial. A further study will be required to determine if the intervention is more effective over time and if that effect is sustained.

## Conclusion

This paper describes the pragmatic conduct of a process evaluation that ran parallel with the conduct of the OptiBIRTH trial. Implementing theoretical interventions in real-world practice settings is complicated and can be imprecise. Adequate testing of complex interventions can be enhanced by parallel process evaluations that monitor and assess the mode of action of an intervention as it is applied in a practice setting. To be able to make accurate interpretations of outcomes, research teams need to know what intervention components were delivered and to what quality and consistency. It is widely accepted that better implementation leads to better outcomes and that multiple factors affect implementation [26]. Therefore, process evaluation is absolutely essential for interpreting findings about the effects of interventions tested in trials, particularly if those interventions are complex. We can conclude from our process evaluation that the OptiBIRTH intervention was delivered during the trial as per the trial protocol.

## Additional file

Additional file 1: Online Apps designed for the OptiBIRTH intervention. (PDF 646 kb)

## Abbreviations

CS: Caesarean section
MOL: Midwife opinion leader
OL: Opinion Leader
OOL: Obstetrician opinion leader
PI: Principal investigator
TIDieR: Template for Intervention Description and Replication
VBAC: Vaginal birth after caesarean section
