# Supplementary material for: Process evaluation for OptiBIRTH, a randomised controlled trial of a complex intervention designed to increase rates of vaginal birth after caesarean section
Source: Trials. 2018 Jan 5;19:9. doi: 10.1186/s13063-017-2401-x (PMC5756437; doi:10.1186/s13063-017-2401-x)
Supplement: Additional file 1: — Online Apps designed for the OptiBIRTH intervention. (PDF 646 kb) [file 13063_2017_2401_MOESM1_ESM.pdf]

BMJ 2014;348:g1687 doi: 10.1136/bmj.g1687 (Published 7 March 2014)

RESEARCH METHODS & REPORTING

Better reporting of interventions: template for intervention description and replication (TIDieR) checklist and guide

Tammy C Hoffmann *associate professor of clinical epidemiology*[1], Paul P Glasziou *director and professor of evidence based medicine*[1], Isabelle Boutron *professor of epidemiology*[2], Ruairidh Milne *professorial fellow in public health and director*[3], Rafael Perera *university lecturer in medical statistics*[4], David Moher *senior scientist*[5], Douglas G Altman *professor of statistics in medicine*[6], Virginia Barbour *medicine editorial director, PLOS*[7], Helen Macdonald *assistant editor*[8], Marie Johnston *emeritus professor of health psychology*[9], Sarah E Lamb *Kadoorie professor of trauma rehabilitation and co-director of Oxford clinical trials research unit*[10], Mary Dixon-Woods *professor of medical sociology*[11], Peter McCulloch *clinical reader in surgery*[12], Jeremy C Wyatt *leadership chair of ehealth research*[13], An-Wen Chan *Phelan scientist*[14], Susan Michie *professor*[15]

[1]Centre for Research in Evidence Based Practice, Faculty of Health Sciences and Medicine, Bond University, Queensland, Australia, 4229; [2]INSERM U738, Université Paris Descartes-Sorbonne Paris Cité, Paris, France; [3]Wessex Institute, University of Southampton, Southampton, UK; [4]Department of Primary Care Health Sciences, University of Oxford, UK; [5]Clinical Epidemiology Program, Ottawa Hospital Research Institute, Ottawa, Canada; [6]Centre for Statistics in Medicine, University of Oxford, UK; [7]PLOS, Brisbane, Australia; [8]BMJ, London, UK; [9]Institute of Applied Health Sciences, University of Aberdeen, Aberdeen, UK; [10]Nuffield Department of Orthopaedics, Rheumatology and Musculoskeletal Sciences, Botnar Research Centre, University of Oxford, Oxford, UK; [11]Department of Health Sciences, University of Leicester, Leicester, UK; [12]Nuffield Department of Surgical Science, University of Oxford, Oxford, UK; [13]Leeds Institute of Health Sciences, University of Leeds, Leeds, UK; [14]Women's College Research Institute, University of Toronto, Toronto, Canada; [15]Centre for Outcomes Research and Effectiveness, Department of Clinical, Educational and Health Psychology, University College London, London, UK

Abstract

Without a complete published description of interventions, clinicians and patients cannot reliably implement interventions that are shown to be useful, and other researchers cannot replicate or build on research findings. The quality of description of interventions in publications, however, is remarkably poor. To improve the completeness of reporting, and ultimately the replicability, of interventions, an international group of experts and stakeholders developed the Template for Intervention Description and Replication (TIDieR) checklist and guide. The process involved a literature review for relevant checklists and research, a Delphi survey of an international panel of experts to guide item selection, and a face to face panel meeting. The resultant 12 item TIDieR checklist (brief name, why, what (materials), what (procedure), who provided, how, where, when and how much, tailoring, modifications, how well (planned), how well (actual)) is an extension of the CONSORT 2010 statement (item 5) and the SPIRIT 2013 statement (item 11). While the emphasis of the checklist is on trials, the guidance is intended to apply across all evaluative study designs. This paper presents the TIDieR checklist and guide, with an explanation and elaboration for each item, and examples of good reporting. The TIDieR checklist and guide should improve the reporting of interventions and make it easier for authors to structure accounts of their interventions, reviewers and editors to assess the descriptions, and readers to use the information.

Introduction

The evaluation of interventions is a major research activity, yet the quality of descriptions of interventions in publications remains remarkably poor. Without a complete published description of the intervention, other researchers cannot replicate or build on research findings. For effective interventions,

Correspondence to: T C Hoffmann thoffmann@bond.edu.au

Extra material supplied by the author (see http://www.bmj.com/content/348/bmj.g1687?tab=related#datasupp)

Appendix 1: Existing checklists and literature used in generation of the Delphi survey items
Appendix 2: Main profession of Delphi survey respondents
Appendix 3: The TIDieR (template for intervention description and replication) checklist
Appendix 4: Examples of different formats that can be used to describe and/or provide study intervention materials

For personal use only: See rights and reprints http://www.bmj.com/permissions Subscribe: http://www.bmj.com/subscribe

clinicians, patients, and other decision makers are left unclear about how to reliably implement the intervention. Intervention description involves more than providing a label or the ingredients list. Key features—including duration, dose or intensity, mode of delivery, essential processes, and monitoring—can all influence efficacy and replicability but are often missing or poorly described. For complex interventions, this detail is needed for each component of the intervention. For example, a recent analysis found that only 11% of 262 trials of cancer chemotherapy provided complete details of the trial treatments.[1] The most frequently missing elements were dose adjustment and "premedications," but 16% of trials omitted even the route of drug administration. The completeness of intervention description is often worse for non-pharmacological interventions: one analysis of trials and reviews found that 67% of descriptions of drug interventions were adequate compared with only 29% of non-pharmacological interventions.[2] A recent study of 137 interventions, from 133 trials of non-drug interventions, found that only 39% of interventions were described adequately in the primary paper or any references, appendices, or websites.[3] This increased, albeit to only 59%, by contacting authors for additional information—a task almost no clinicians and few researchers have time to undertake.

The Consolidated Standards of Reporting Trials (CONSORT) 2010 statement[4] currently suggests in item 5 that authors should report on "The interventions for each group with sufficient details to allow replication, including how and when they were actually administered." This is appropriate advice, but further guidance seems to be needed: despite endorsement of the CONSORT statement by many journals, reporting of interventions is deficient. The problem arises partly from lack of awareness among authors about what comprises a good description and partly from lack of attention by peer reviewers and editors.[5]

A small number of CONSORT extension statements contain expanded guidance about describing interventions, such as non-pharmacological interventions,[6] and specific categories of interventions, such as acupuncture and herbal interventions.[7 8] The guidance for content of trial protocols, SPIRIT (Standard Protocol Items: Recommendations for Interventional Trials), provides some recommendations for describing interventions in protocols.[9] More generic and comprehensive guidance is needed along with robust ways to implement such guidance. We developed an extension of item 5 of the CONSORT 2010 statement and item 11 of the SPIRIT 2013 statement in the form of a checklist and guidance entitled TIDieR (Template for Intervention Description and Replication), with the objective of improving the completeness of reporting, and ultimately the replicability, of interventions. This article describes the methods used to develop and obtain consensus for this checklist and, for each item, provides an explanation, elaboration, and examples of good reporting. While the emphasis of the checklist is on trials, the guidance is intended to apply across all evaluative study designs, such as trials, case-control studies, and cohort studies.

Methods for development of the TIDieR checklist and guide

Development of the checklist followed the methodological framework for developing reporting guidelines suggested by the EQUATOR Network.[10] In collaboration with the CONSORT steering group, we established a TIDieR steering committee (PPG, TCH, IB, RM, RP). The committee generated a list of 34 potential items from relevant CONSORT checklists and checklists for reporting discipline-specific or particular categories of interventions. The group also reviewed other sources of guidance on intervention reporting identified from a thorough search of the literature, followed by a forward and backward citation search (see appendix 1).

We then used a two round modified Delphi consensus survey method[11] involving a broad range of expertise and stakeholders. In the first round, each of the 34 items generated by the steering committee was rated by survey participants as "omit," "possible," "desirable," or "essential" to include in the final checklist. From the first round, some items were reworded and combined, and then the ranked items were divided into three groups for the second round. The first group contained 13 items with the highest rankings (rated as "essential" by ≥70% participants or "essential or desirable" by ≥85%), and participants were advised that these would be included in the checklist unless strong objection to their inclusion was received in the second round. The second group contained 13 items with moderate rankings ("essential or desirable" by ≥65%); participants were asked to rate each of these again as "omit," "possible," "desirable," or "essential." The third group contained three items with low rankings, and participants were advised that these items would be removed unless strong objection to their omission was received in the second round. In both rounds, participants could also suggest additional items, comment on item wording, or provide general comments.

Delphi participants (n=125) were authors of research on describing interventions, clinicians, authors of existing reporting guidelines, clinical trialists, methodologists or statisticians with expertise in clinical trials, and journal editors (see appendix 2). They were invited by email to complete the two rounds of the web based survey. The response rate was 72% (n=90) for the first round. Only those who completed round one and were willing to participate in round two were invited to participate in round two. The response rate for round two was 86% (74 of 86 invited).

After the two Delphi rounds, 13 items were included in the draft checklist, and 13 moderately rated items were retained for further discussion at the in person meeting. The results of the Delphi survey were reported at a two day consensus meeting on 27-28 March 2013, in Oxford, UK. Thirteen invited experts, representing a range of health disciplines (see author list) and with expertise in the development of trial, methodological, and/or reporting guidelines, attended and are all authors of this paper. The meeting began with a review of the literature on intervention reporting, followed by a report of the Delphi process, the draft checklist of 13 items, and rankings of and comments about the additional 13 moderately rated items. Meeting participants discussed the proposed items and agreed which should be included and the wording of each item.

After the meeting, the checklist was distributed to the participants to ensure it reflected the decisions made, and this explanation and elaboration document was drafted. This was then piloted with 26 researchers who were authoring papers of intervention studies and minor clarifications were made in the elaboration of some items.

Scope of the TIDieR checklist and guide for describing interventions

The overarching purpose of the TIDieR checklist is to prompt authors to describe interventions in sufficient detail to allow their replication. The checklist contains the minimum recommended items for describing an intervention. Authors

For personal use only: See rights and reprints http://www.bmj.com/permissions Subscribe: http://www.bmj.com/subscribe

should provide additional information where they consider it necessary for the replication of an intervention.

Most TIDieR items are relevant for most interventions and applicable to even apparently simple drug interventions, which are sometimes poorly described.[2] If we consider the elements of an evaluation of an intervention—the population, intervention, comparison, outcome ("PICO")—TIDieR can be seen as a guide for reporting the intervention and comparison (and co-interventions, when relevant) elements of a study. Other elements (such as population, outcomes) and methodological features are covered by CONSORT 2010 or SPIRIT 2013 items for randomised trials and by other checklists (such as the STROBE statement[12]) for alternate study designs. They have not been duplicated as part of the TIDieR checklist.

The order in which items are presented in the checklist does not necessarily reflect the order in which information should be presented. It might also be possible to combine a number of items from the checklist into one sentence. For example, information about what materials (item 3) and what processes (item 4) can be combined (example 3c).

We emphasise that our definition of "intervention" extends to describing the intervention received by the comparison group/s in a study. Control interventions and co-interventions are often particularly poorly described; "usual care" is not a sufficient description. When a controlled study is reported, authors should describe what participants in the control group received with the same level of detail used to describe the intervention group, within the limits of feasibility. Full understanding of the comparison group care can help to explain the observed efficacy of an intervention, with greater apparent effect sizes being potentially found when control group care is minimal.[13] Describing the care that each group received will usually require the replication of the checklist for each group in a study.

As well as describing which interventions (or control conditions) were delivered to different groups, authors should also explain legitimate variants of the intervention. Authors might find it helpful to locate their trial on the pragmatic explanatory continuum.[14] If, for example in a pragmatic trial, authors expect there to be variants in aspects of the intervention (for instance, in the "usual care" group across various centres), those variants should be described under the appropriate checklist items.

We recognise that limitations (such as format and length) for journals that are only paper based can sometimes preclude inclusion of all intervention information in the primary paper (that is, the paper that is reporting the main results of the intervention evaluation). The information that is prompted by the TIDieR checklist might therefore be reported in locations beyond the primary paper itself, including online supplementary material linked to the primary paper, a published protocol and/or other published papers, or a website. Authors should specify the location of additional detail in the primary paper (for example, "online appendix 2 for the training manual," "available at www...," or "details are in our published protocol"). When websites provide further details, URLs that are designed to remain stable over time are essential.

The TIDieR checklist explanation and elaboration

The items included in the checklist are shown in table 1⇓. The complete checklist is available in appendix 3 and a Word version, which authors and reviewers can fill out, is available on the EQUATOR Network website (www.equator-network.org/reporting-guidelines/tidier/). An explanation for each item is given below, along with examples of good reporting. Citations for the examples are in table 2.⇓

Item 1. Brief name: Provide the name or a phrase that describes the intervention

Examples:

- 1a. Single . . . dose of dexamethasone
- 1b. TREAD (TREAtment of Depression with physical activity) study
- 1c. Internet based, nurse led vascular risk factor management programme promoting self management

Explanation—Precision in the name, or brief description, of an intervention enables easy identification of the type of intervention and facilitates linkage to other reports on the same intervention. Give the intervention name (examples 1a, 1b), explaining any abbreviations or acronyms in full (example 1b), or a short (one or two line) statement of the intervention without elaboration (example 1c).

Item 2. Why: Describe any rationale, theory, or goal of the elements essential to the intervention

Examples:

- 2a. Dexamethasone (10 mg) or placebo was administered 15 to 20 minutes before or with the first dose of antibiotic. . . Studies in animals have shown that bacterial lysis, induced by treatment with antibiotics, leads to inflammation in the subarachnoid space, which may contribute to an unfavourable outcome [references]. These studies also show that adjuvant treatment with anti-inflammatory agents, such as dexamethasone, reduces both cerebrospinal fluid inflammation and neurologic sequelae [references]
- 2b. Self management of oral anticoagulant therapy may result in a more individualised approach, increased patient responsibility, and enhanced compliance, which may lead to improvement in the regulation of anticoagulation
- 2c. The TPB [Theory of Planned Behaviour] informed the hypothesised mediators of intention and physical activity that were targeted in the intervention program: instrumental and affective attitude, subjective norm and perceived behavioural control
- 2d. We chose a 5° wedge because greater wedging is less likely to be tolerated by the wearer [reference] and is difficult to accommodate within a normal shoe

Explanation—Inclusion of the rationale, theory, or goals that underpin an intervention, or the components of a complex intervention,[15] can help others to know which elements are essential, rather than optional or incidental. For example, the colour of capsules used in a pharmacological intervention is likely to be an incidental, not essential, contributor to the intervention's efficacy and hence reporting of this is not necessary. In some reports, the term "active ingredient" is used and refers to the components within an intervention that can be specifically linked to its effect on outcomes such that, if they were omitted, the intervention would be ineffective.[16] The known or supposed mechanism of action of the active component/s of the intervention should be described.

Example 2a illustrates the rationale for treating bacterial meningitis with dexamethasone in addition to an antibiotic. Behaviour change and implementation interventions might require different forms of description, but the basic principles are the same. It might, alongside an account of the components

of the intervention, also be appropriate to describe the intervention in terms of its theoretical basis, including its hypothesised mechanisms of action (examples 2b, 2c).[17-19] The rationale behind an important element of an intervention can sometimes be pragmatic and relate to acceptability of the intervention by participants (example 2d).

Item 3. What (materials): Describe any physical or informational materials used in the intervention, including those provided to participants or used in intervention delivery or in training of intervention providers. Provide information on where the materials can be accessed (for example, online appendix, URL)

Examples:

- 3a. The educational package included a 12-minute cartoon . . . The presentation of the cartoon was complemented by classroom discussions, display of the same poster that was used for the control group [see figure in appendix 4], dissemination of a pamphlet summarising the key messages delivered in the cartoon, and drawing and essay writing competitions to reinforce the messages . . . The cartoon can be accessed at NEJM.org or at [URL provided]. A specific teacher training workshop was held before commencement of the trial (for details, see the protocol, available at NEJM.org)
- 3b. The intervention group received a behaviour change counselling training programme called the Talking Lifestyle learning programme that took practitioners through a portfolio-driven set of learning activities. Precise details of both intervention content and the training programme can be found in [URL, login and password provided]. . . Box 1 provides a more detailed description of the components of the training programme
- 3c. The "local" group received a sonographically guided injection of 2 mL (10 mg/mL) triamcinolone (Kenacort-T, Bristol-Myers Squibb) and 5 mL (10 mg/mL) lidocaine hydrochloride (Xylocaine, AstraZeneca) to the subacromial bursa and an intramuscular injection of 4 mL (10 mg/mL) lidocaine hydrochloride to the upper gluteal region

Explanation—A full description of an intervention should describe what different physical and information materials were used as part of the intervention (this typically will not extend to study consent forms unless they provide written instructions about the intervention that are not provided elsewhere). Intervention materials are the most commonly missing element of intervention descriptions.[3] This list of materials can be regarded as comparable with the "ingredients" required for a recipe. It can include materials provided to participants (example 3a), training materials used with the intervention providers (examples 3a, 3b), or the surgical device or pharmaceutical drug used and its manufacturer (example 3c). For some interventions, it might be possible to describe the materials and the procedures (item 4) together (examples 3c, 4c). If the information is too long or complex to describe in the primary paper, alternative options and formats for providing the materials should be used (see appendix 4 for some examples) and details of where they can be obtained (examples 3a, 3b) should be provided in the primary paper.

Item 4. What (procedures): Describe each of the procedures, activities, and/or processes used in the intervention, including any enabling or support activities

Examples:

- 4a. The TREPP [transrectus sheath preperitoneal] technique can be performed under spinal anaesthesia. To reach the PPS [preperitoneal space], a 5 cm straight incision is made about 1 cm above the pubic bone. The anterior rectus sheath is opened, as is the underlying fascia transversalis [figure]. After retraction of the muscle fibres medially, the inferior epigastric vein and artery are identified and retracted medially as well
- 4b. . . . identified a suitable vein for cannulation. The overlying skin was wiped with an alcohol swab and allowed to dry, as per standard operating procedures. The principal investigator then administered the allocated spray from a distance of about 12 cm for two seconds. This technique avoided "frosting up" of vapocoolant on the skin. Liquid spray on the skin was allowed to evaporate for up to 10 seconds. The area was again wiped with an alcohol swab and cannulation proceeded immediately. Cannulation had to be carried out within 15 seconds of administration of the spray
- 4c. . . . three periods of exercise each lasting 5 min, supervised by a physiotherapist. The first period consisted of 2 min of indoor jogging, 1 min of stair climbing (three floors), and 2 min of cycling on an ergometer. Resistance on the ergometer was adjusted to ensure that the participant's respiratory rate was elevated during the 2 min of cycling. At the end of the first period, the patient performed several prolonged and brief expiratory flow accelerations with open glottis, the forced expiratory technique, and finally cough and sputum expectoration. These clearance manoeuvres were performed over 1.5 min. The second period consisted of 1 min of stretching repeated five times, followed by the same expiratory manoeuvres for 1.5 min, as described above. The third period consisted of continuous jumping on a small trampoline. It included 2 min of jumping, 2 min of jumping while throwing and catching a ball, and 1 min of jumping while hitting a tossed ball. This was again followed by expiratory manoeuvres for 1.5 min. The entire regimen was followed by 40 min rest
- 4d. All health workers doing outpatient consultations in the intervention group received text messages about malaria case management for 6 months . . . The key messages addressed recommendations from the Kenyan national malaria guidelines and training manuals [references]
- 4e. Onsite activities were implemented by hospital personnel responsible for quality improvement initiatives . . . Standard communication channels were used, including group specific computer based training modules and daily electronic documentation by nursing staff for all groups. On-site training in bathing with chlorhexidine-impregnated cloths was provided to hospitals assigned to a decolonisation regimen . . . Nursing directors performed at least three quarterly observations of bathing, including questioning staff about protocol details. Investigators hosted group specific coaching teleconferences at least monthly to discuss implementation, compliance, and any new potentially conflicting initiatives

Explanation—Describe what processes, activities, or procedures the intervention provider/s carried out. Continuing the recipe metaphor used above, this item refers to the "methods" section

For personal use only: See rights and reprints http://www.bmj.com/permissions Subscribe: http://www.bmj.com/subscribe

of a recipe and where intervention materials ("ingredients") are involved, describes what is to be done with them. "Procedure" can refer to the sequence of steps to be followed (examples 3c, 4b) and is a term used by some disciplines, particularly surgery, and includes, for example, preoperative assessment, optimisation, type of anaesthesia, and perioperative and postoperative care, along with details of the actual surgical procedure used (example 4a). Examples of processes or activities include referral, screening, case finding, assessment, education, treatment sessions (example 4c), telephone contacts (example 4d), etc. Some interventions, particularly complex ones, might require additional activities to enable or support the intervention to occur (in some disciplines these are known as implementation activities), and these should also be described (example 4e). Elaboration about how to report interventions where the procedure is not the same for all participants is provided at item 9 (tailoring).

Item 5. Who provided: For each category of intervention provider (for example, psychologist, nursing assistant), describe their expertise, background and any specific training given

Examples:

- 5a. Only female counsellors were included in this rural area, after consultation with the village chiefs, because it would not have been deemed culturally appropriate for men to counsel women without their husband present . . . Selection criteria for lay counsellors included completion of 12 years of schooling, residence in the intervention area, and a history of community work
- 5b. The procedure is simple, uses existing surgical skills, and has a short learning curve, with the manufacturers recommending at least five mentored cases before independently practising. All surgeons involved in the study will have completed this training and will have carried out over five procedures prior to recruiting to the study
- 5c. Therapists received at least one day of training specific to the trial from an experienced CBT [cognitive behaviour therapy] therapist and trainer and weekly supervision from skilled CBT supervisors at each centre. . . The intervention was delivered by 11 part time therapists in the three sites who were representative of those working within NHS psychological services [reference]. Ten of the 11 therapists were female, their mean age was 39.2 years (SD 8.1), and they had practised as a therapist for a mean of 9.7 years (8.1) . . . Nine of the 11 therapists delivered 97% of the intervention and, for these nine, the number of patients per therapist ranged from 13 (6%) to 41 (18%)
- 5d. . . . brief lifestyle counselling was practised with trained actors and tape recorded. The competency of counselling was checked using the behaviour change counselling index [reference]. Only practitioners who reached a required standard (agreed by inter-rater consensus between three independent clinical assessors) were approved to deliver brief lifestyle counselling in the trial

Explanation—The term "intervention provider" refers to who was involved in providing the intervention (for example, by delivering it to recipients or undertaking specific tasks). This is important in circumstances where the providers' expertise and other characteristics (example 5a) could affect the outcomes of the intervention. Important issues to address in the description might include the number of providers involved in delivering or undertaking the intervention; their disciplinary background (for example, nurse, occupational therapist, colorectal surgeon, expert patient); what pre-existing specific skills, expertise, and experience providers required and if and how these were verified; details of any additional training specific to the intervention that needed to be given to providers before (example 3b) and/or during the study (example 5c); and if competence in delivering the intervention was assessed before (example 5d) or monitored throughout the study and whether those deemed lacking in competence were excluded (example 5d) or retrained. Other information about providers could include whether the providers were doing the intervention as part of their normal role (example 3b) or were specially recruited as providers for purposes of the study (example 5c); whether providers were reimbursed for their time or provided with other incentives (if so, what) to deliver the intervention as part of the study, and whether such time or incentives might be needed to replicate the intervention.

Item 6. How: Describe the modes of delivery (such as face to face or by some other mechanism, such as internet or telephone) of the intervention and whether it was provided individually or in a group

Examples:

- 6a. . . . sessions . . . held weekly and facilitated in groups of 6-12 by . . .
- 6b. Drugs were delivered by . . . members of the [Reproductive and Child Health] trekking teams . . . teams visited each of the study villages . . .
- 6c. The text messaging intervention, SMS Turkey, provided six weeks of daily messages aimed at giving participants skills to help them quit smoking. Messages were sent in an automated fashion, except two days and seven days after the initial quit day
- 6d. . . . made their own appointments online . . . Participants and therapists typed free text into the computer, with messages sent instantaneously; no other media or means of communication were used
- 6e. . . . three 1 hour home visits (televisits) by a trained assistant . . . ; participants' daily use of an in-home messaging device . . .. . that was monitored weekly by the teletherapist; and five telephone intervention calls between the teletherapist and the participant . . .

Explanation—Specify whether the intervention was provided to one participant at a time (such as a surgical intervention) or to a group of participants and, if so, the group size (example 6a). Also describe whether it was delivered face to face (example 6b), by distance (such as by telephone, surface mail, email, internet, DVD, mass media campaign, etc) as in examples 6c, 6d, or a combination of modes (example 6e). When relevant, describe who initiated the contact (example 6c), and whether the session was interactive (example 6d) or not (example 6c), and any other delivery features considered essential or likely to influence outcome.

Item 7. Where: Describe the type(s) of location(s) where the intervention occurred, including any necessary infrastructure or relevant features

Examples:

- 7a. . . . medication . . . and a spacer (as appropriate) were delivered to the school nurse for directly observed therapy

For personal use only: See rights and reprints http://www.bmj.com/permissions Subscribe: http://www.bmj.com/subscribe

on the days on which the child attended school. . . An additional canister of preventive medication was delivered to the child's home to use on weekends and other days the child did not attend school, and the child's caregiver was shown proper administration technique

- 7b. Women were recruited from three rural and one peri-urban antenatal clinic in Southern Malawi . . . tablets were taken under supervision at the clinic
- 7c. . . . participants for the . . telehealth trial, across three sociodemographically distinct regions in England (rural Cornwall, rural and urban Kent, and urban Newham in London) comprising four primary care trusts. . . Control participants had no telehealth or telecare equipment installed their homes for the duration of the study. A Lifeline pendant (a personal alarm) plus a smoke alarm linked to a monitoring centre were not, on their own, sufficient to classify as telecare for current purposes
- 7d. Most births in African countries occur at home, especially in rural areas . . . They identified pregnant women and made five home visits during and after pregnancy . . . Peer counsellors lived in the same communities, so informal contacts to make arrangements for visits were common. . . counsellors were . . . given a bicycle, T shirt. . .
- 7e. This paper contains a box, titled "Key features of healthcare systems in Northern Ireland and Republic of Ireland," which summarises relevant aspects of general practices such as funding, registration, and access to free prescriptions

Explanation—In some studies the intervention can be delivered in the same location where participants were recruited and/or data were collected and details might therefore already be included in the primary paper (for example, as in item 4b of CONSORT 2010 statement if reporting a trial). If, however, the intervention occurred in different locations, this should be specified. At its simplest level, the location might be, for example, in the participants' home (example 7a), residential aged care facility, school (example 7a), outpatient clinic (example 7b), inpatient hospital room, or a combination of locations (example 7a). Features or circumstances about the location can be relevant to the delivery of the intervention and should be described (examples 7e). For example, they might include the country (example 7b), type of hospital or primary care (example 7c), publicly or privately funded care, volume of activity, details of the healthcare system, or the availability of certain facilities or equipment (examples 7c, 7d, 7e). These features can impact on various aspects of the intervention such as its feasibility (example 7d) or provider or participant adherence and are important for those considering replicating the intervention.

Item 8. When and how much: Describe the number of times the intervention was delivered and over what period of time including the number of sessions, their schedule, and their duration, intensity or dose

Examples:

- 8a. . . . a loading dose of 1 g of tranexamic acid infused over 10 min, followed by an intravenous infusion of 1 g over 8 h
- 8b. They received five text messages a day for the first five weeks and then three a week for the next 26 weeks
- 8c. . . . . exercise three times a week for 24 weeks. . . Participants began with 15 minutes of exercise and increased to 40 minutes by week eight . . . Between weeks eight and 24, attempts to increase exercise intensity were made at least weekly either by increasing treadmill speed or by increasing the treadmill grade. Participants with leg symptoms were encouraged to exercise to near maximal leg symptoms. Asymptomatic participants were encouraged to exercise to a level of 12 to 14 . . . . on the Borg rating of perceived exertion scale [reference]
- 8d. . . . delivered weekly one hour sessions in the woman's home, for up to eight weeks . . . starting at around eight weeks postnatally

Explanation—The type of information needed about the "when and how much" of the intervention will differ according to the type of intervention. For some interventions some aspects will be more important than others. For example, for pharmacological interventions, the dose and scheduling is often important (example 8a); for many non-pharmacological interventions, the "how much" of the intervention is instead described by the duration and number of sessions (examples 8b, 8c). For multiple session interventions, the schedule of the sessions is also needed (example 8b) and if the number of sessions, their schedule, and/or intensity was fixed (examples 8b, 4c, 6a) or if it could be varied according to rules and if so, what they were (example 8c). Tailoring of the intervention to individuals or groups of individuals is elaborated on in item 9 (tailoring). For some interventions, as part of the "when" information, detail about the timing of the intervention in relation to relevant events might also be important (for example, how long after diagnosis, first symptoms, or a crucial event did the intervention start) (example 8d). As described below in item 12, the "amount" or dose of intervention that participants actually received might differ from the amount intended. This detail should be described, usually in the results section (examples 12a-c).

Item 9. Tailoring: If the intervention was planned to be personalised, titrated or adapted, then describe what, why, when, and how

Examples:

- 9a. Those allocated to the intervention arm followed an intensive stepped programme of management, with mandatory visits to their doctor at weeks 6, 10, 14, and 18 after randomisation to review their blood pressure and to adjust their treatment if needed according to prespecified algorithms [provided in supplementary appendix]
- 9b. All patients received laparoscopic mini-gastric bypass surgery. . . The bypass limb was adjusted according to the preoperative BMI of the patient. A 150 cm limb was used for BMI 35, with a 10 cm increase in the bypass limb with every BMI category increase, instead of using a fixed limb for all patients
- 9c. Participants began exercising at 50% of their 1 rm [repetition maximum]. Weights were increased over the first five weeks until participants were lifting 80% of their 1 rm. Weights were adjusted after each monthly 1 rm and as needed to achieve an exercise intensity of a rating of perceived exertion of 12 to 14
- 9d. Stepped-care decisions for patients . . . were guided by responses to the nine item patient health questionnaire [reference], administered at each treatment visit and formally evaluated at eight week intervals. Patients who

For personal use only: See rights and reprints http://www.bmj.com/permissions Subscribe: http://www.bmj.com/subscribe

did not show prespecified improvement were offered the choice of switching treatments (for example, from problem solving therapy to medication), adding the other treatment, or intensifying the original treatment choice, based on the treatment team's recommendation (for details, see [reference])

Explanation—In tailored interventions, not all participants receive an identical intervention. Interventions can be tailored for several reasons, such as titration to obtain an appropriate "dose" (example 9a); participant's preference, skills, or situation (example 9b); or it may be an intrinsic element of the intervention as with increasing intensity of an exercise (example 9c). Hence, a brief rationale and guide for tailoring should be provided, including any variables/constructs used for participant assessment (examples 9b, 9c) and subsequent tailoring. Tailoring can occur at several stages and authors should describe any decision points and rules used at each point (example 9d). If any decisional or instructional materials are used, such as flowcharts, algorithms or dosing nomograms, these should be included, referenced (example 9d), or their location provided (example 9a).

Item 10. Modifications: If the intervention was modified during the course of the study, describe the changes (what, why, when, and how)

Examples:

- 10a. A mixture of general practitioners and practice care nurses delivered 95% of screening and brief intervention activity in this trial. . . Owing to this slow recruitment, research staff who had delivered training in study procedures supported screening and brief intervention delivery in 10 practices and recruited 152 patients, which was 5% of the total number of trial participants
- 10b. Computers with slow processing units and poor internet connections meant that seven general practitioners never got functional software; they used a structured paper version that was faxed between the research team and general practitioner after each appointment

Explanation— This item refers to modifications that occur at the study level, not individual tailoring as described in item 9. Unforeseen modifications to the intervention can occur during the course of the study, particularly in early studies. If this happens, it is important to explain what was modified, why and when modifications occurred, and how the modified intervention differed from the original (example 10a—modification to who provided the intervention; example 10b— modification in the materials). Modifications sometimes reflect changing circumstances. In other studies, they can show learning about the intervention, which is important to transmit to the reader and others to prevent unnecessary repetition of errors during attempts to replicate the intervention. If changes to the intervention occurred between the published protocol or published pilot study and the primary paper, these changes should also be described.

Item 11. How well (planned): If intervention adherence or fidelity was assessed, describe how and by whom, and if any strategies were used to maintain or improve fidelity, describe them

Examples:

- 11a. Pathologists were trained to identify lateral spread of tumour according to the protocol [reference]. The results of histopathological examination of the specimens were reviewed by a panel of supervising pathologists and a quality manager
- 11b. Staff in the study sites were trained initially, and therapy supervision was provided by weekly meetings between therapists and investigators. Cognitive therapy sessions were taped with the participant's consent so that participants could be asked to listen to the tapes as part of their homework and to assist supervision. During the course of the trial a sample of 80 tapes was rated according to the cognitive therapy scale-revised [reference] and the cognitive therapy for at risk populations adherence scale [reference] to ensure rigorous adherence to the protocol throughout the duration of the trial. These tapes were drawn from both early and late phases of therapy and included participants from each year of recruitment
- 11c. Adherence to trial medication was assessed by means of self reported pill counts collected during follow-up telephone calls. These data were categorised as no pills taken, hardly any taken (1-24% of prescribed doses), some taken (25-49%), most taken (50-74%), or all taken (75-100%)
- 11d. Training will be delivered independently in each of the three regional study centres. All trainers will adhere to a single training protocol to ensure standardised delivery of the training across centres. Training delivery will be planned and rehearsed jointly by all trainers using role play and peer review techniques. In addition, the project manager will act as an observer during the first two training sessions in each centre and will provide feedback to trainers with a view to further standardising the training [note, this example is from a protocol]

Explanation—Fidelity refers to the degree to which an intervention happened in the way the investigators intended it to[20] and can affect the success of an intervention.[21] The terms used to describe this concept vary among disciplines and include treatment integrity, provider or participant adherence, and implementation fidelity. This item—and item 12—extends beyond simple receipt of the intervention (such as how many participants were issued with the intervention drug or exercises) and refers to "how well" the intervention was received or delivered (such as how many participants took the drug/did the exercises, how much they took/did, and for how long). Depending on the intervention, fidelity can apply to one or more parts of the intervention, such as training of providers (examples 11a, 11b, 11d), delivery of the intervention (example 11b), and receipt of the intervention (example 11c). The types of measures used to determine intervention fidelity will also vary according to the type of intervention. For example, in simple pharmacological interventions, assessing fidelity often focuses on recipients' adherence to taking the drug (example 11b). In complex interventions, such as rehabilitation, psychological, or behaviour change interventions, however, assessment of fidelity is also more complex (example 11b). There are various preplanned strategies and tools that can be used to maintain fidelity before delivery of the intervention (example 11d) or during the study (example 11b). If any strategies or tools were used to maintain fidelity, they should be clearly described. Any materials used as part of assessing or maintaining fidelity should be included, referenced, or their location provided.

Item 12: How well (actual): If intervention adherence or fidelity was assessed, describe

For personal use only: See rights and reprints http://www.bmj.com/permissions Subscribe: http://www.bmj.com/subscribe

the extent to which the intervention was delivered as planned

Examples:

- 12a. The mean (SD) number of physiotherapy sessions attended was 7.5 (1.9). Seven patients (9%) completed less than four physiotherapy sessions; the reasons included non-attendance, moving interstate, or recovery from pain. Of patients in the physiotherapy groups, 70% were compliant with their home exercise program during at least five of seven weeks
- 12b. The EE [early exercise] group reported an adherence rate of 73% at [time] T2 and 75.7% at [time] T3, and the CE [delayed exercise] group reported 86.7% adherence at T3 . . . with the early exercise EE group reporting disease and treatment related barriers to exercise during their cancer treatment (“week of chemotherapy” 14%; “fatigue” 10%); or life related barriers (“illness eg, colds or flu” 16%; “family obligations” 13%)”
- 12c. A total of 214 participants (78%) reported taking at least 75% of the study tablets; the proportion of patients who reported taking at least 75% of the tablets was similar in the two groups
- 12d. The integrity of the psychological therapy was assessed with the cognitive therapy rating scale [reference] to score transcripts of 40 online sessions for patients who had completed at least five sessions of therapy. With use of computer generated random numbers, at least one such patient was selected for each therapist. For these patients, either session six or the penultimate session was rated by two independent CBT [cognitive behaviour therapy]-trained psychologists, who gave mean ratings of 31 (SD between therapists 9) and 32 (13) of 72

Explanation— For various reasons, an intervention, or parts of it, might not be delivered as intended, thus affecting the fidelity of the intervention. If this is assessed, authors should describe the extent to which the delivered intervention varied from the intended intervention. This information can help to explain study findings, minimise errors in interpreting study outcomes, inform future modifications to the intervention, and, when fidelity is poor, can point to the need for further studies or strategies to improve fidelity or adherence.[22 23] For example, there might be some aspects of the intervention that participants do not like and this could influence their adherence. The way in which the intervention fidelity is reported will reflect the measures used to assess it (examples 12a-d), as described in item 11.

Discussion

Who should use TIDieR?

We describe a short list of items that we believe can be used to improve the reporting of interventions and make it easier for authors to structure accounts of their interventions, reviewers and editors to assess the descriptions, and readers to use the information. Consistent with the CONSORT 2010 and SPIRIT 2013 statements, we recommend that interventions are described in enough detail to enable replication, and recommend that authors use the TIDieR checklist to achieve this. As inclusion of all intervention details is not always possible in the primary paper of a study, the TIDieR checklist encourages authors to indicate that they have reported each of the items and to state where this information is located (see appendix 3).

The number of checklist items reported is improved when journals require checklist completion as part of the submission process.[24] We encourage journals to endorse the use of the TIDieR checklist, in a similar way to CONSORT and related statements. This can be done by modifying their author instructions, publishing an editorial about intervention reporting, and including a link to the checklist on their website. Few journals currently provide specific guidance about how to report interventions.[25] A small number have editorial policies stating that they will not publish trials unless intervention protocols or full details are available.[26] We encourage other journals to consider adopting similar policies. Any links provided by journals and authors should be reliable and enduring. Stable depositories for descriptions of interventions are also required, and their development needs the contribution and collaboration of all stakeholders in the research community (such as researchers, journal editors, publishers, research funding bodies).

Authors might also want to be guided by the TIDieR items when describing interventions in systematic reviews so that readers of reviews have access to full details of any intervention (or at least details about where to obtain further information) that they want to replicate after reading the review.

Using TIDieR in conjunction with the CONSORT and SPIRIT Statements

For authors submitting reports of randomised trials, we suggest using the TIDieR in conjunction with the CONSORT checklist: when authors complete item 5 of the CONSORT checklist, they should insert “refer to TIDieR checklist” and provide a separate completed TIDieR checklist. For journals that adopt this recommendation, their instructions to authors will need to be modified accordingly and their editors and reviewers made aware of the change. Similarly, for authors submitting protocols of trials, the TIDieR checklist can be referred to when dealing with item 11 of the SPIRIT 2013 checklist. One point of difference is that two TIDieR items (items 10 and 12) are not applicable to intervention reporting in protocols because they cannot be completed until the study is complete. This is noted on the TIDieR checklist. Published protocols are likely to grow in importance as a source of information about the intervention and use of TIDieR in conjunction with the SPIRIT 2013 statement can facilitate this. For authors of study designs other than randomised trials, TIDieR can be used alone as a standalone checklist or in conjunction with the relevant statement for that study design (such as the STROBE statement[12]). We acknowledge that describing complex interventions well can be challenging and that for some particularly complex interventions, a checklist, such as TIDieR, could go some way towards assisting with intervention reporting but might not be able to capture the full complexity of these interventions.

We recognise that adhering to the TIDieR checklist might increase the word count of a paper, particular if the study protocol is not publicly available. We believe this might be necessary to help improve the reporting of studies generally and interventions specifically. As journals recognise the importance of well reported studies and fully described methods, and many move to a model of online only, or a hybrid of printed and online with posting of the full study protocol, this might become less of a barrier to quality reporting. For example, the Nature Publishing Group recently removed word limits on the methods section of submitted papers and advises that: “If more space is required to describe the methods completely, the author should include the 300-word section ‘Methods Summary’ and provide an additional ‘Methods’ section at the end of the text, following the figure legends. This Methods section will appear in the

For personal use only: See rights and reprints http://www.bmj.com/permissions Subscribe: http://www.bmj.com/subscribe

online . . . version of the paper, but will not appear in the printed issue. The Methods section should be written as concisely as possible but should contain all elements necessary to allow interpretation and replication of the results."[27]

Conclusion

The TIDieR checklist and guide should assist authors, editors, peer reviewers, and readers. Some authors might perceive this checklist as another time consuming hurdle and elect to seek publication in a journal that does not endorse reporting guidelines. There is a large evidence base indicating that the quality of reporting of health research is unacceptably poor. Properly endorsed and implemented reporting guidelines offer a way for publishers, editors, peer reviewers, and authors to do a better job of completely and transparently describing what was done and found.[28] Doing so will help reduce wasteful research[29 30] and increase the potential impact of research on health.

We are grateful to everyone who responded to the Delphi survey and for their thoughtful comments. We also thank Nicola Pidduck (Department of Primary Care Health Sciences, Oxford University) for her assistance in organising the consensus meeting in Oxford.

Contributors: PPG and TCH initiated the TIDieR group and led the organising of the Delphi survey and consensus meeting, in conjunction with the other members of the steering group (IB, RM, and RP). TCH led the writing of the paper. All authors contributed to the drafting and revision of the paper and approved the final version. TCH and PPG are guarantors.

Funding: There was no explicit funding for the development of this checklist and guide. The consensus meeting in March 2013 was partially funded by a NIHR Senior Investigator Award held by PPG. TCH is supported by a National Health and Medical Research Council of Australia (NHMRC)/Primary Health Care Research Evaluation and Development Career Development Fellowship (1033038) with funding provided by the Australian Department of Health and Ageing. PPG is supported by a NHMRC Australia Fellowship (527500). DGA is supported by a programme grant from Cancer Research UK (C5529). MDW is supported by a Wellcome Trust Senior Investigator award (WT097899MA).

Competing interests: All authors have completed the Unified Competing Interest form at www.icmje.org/coi_disclosure.pdf (available on request from the corresponding author) and declare: RM is employed by NETSCC, part of the National Institute for Health Research (NIHR) in England. NETSCC manages on behalf of NIHR the NIHR Journals Library, "a suite of five open access journals providing an important and permanent archive of research funded by the National Institute for Health Research." The NIHR Journals Library places great value on reporting the full results of funded research and so is likely to be a user of TIDieR, as it is of other reporting guidelines. VB was the Chief Editor of PLOS Medicine at the time of the consensus meeting and initial drafting of this paper. HM is an assistant editor at BMJ but was not involved in any decision making regarding this paper.

Provenance and peer review: Not commissioned; externally peer reviewed.

1 Duff J, Leather H, Walden E, LaPlant K, George T. Adequacy of published oncology randomised controlled trials to provide therapeutic details needed for clinical application. *J Natl Cancer Inst* 2010;102:702-5.
2 Glasziou P, Meats E, Heneghan C, Shepperd S. What is missing from descriptions of treatment in trials and reviews? *BMJ* 2008;336:1472-4.
3 Hoffmann T, Erueti C, Glasziou P. Poor description of non-pharmacological interventions: analysis of consecutive sample of randomised trials. *BMJ* 2013;347:f3755.
4 Schulz K, Altman D, Moher D, CONSORT Group. CONSORT 2010 Statement: updated guidelines for reporting parallel group randomised trials. *BMJ* 2010;340:c332.
5 Schroter S, Glasziou P, Heneghan C. Quality of descriptions of treatments: a review of published randomised controlled trials. *BMJ Open* 2012;2:e001978.
6 Boutron I, Moher D, Altman D, Schulz K, Ravaud P. Extending the CONSORT statement to randomised trials of nonpharmacologic treatment: explanation and elaboration. *Ann Intern Med* 2008;148:295-310.
7 MacPherson H, Altman DG, Hammerschlag R, Youping L, Taixiang W, White A, et al. Revised standards for reporting interventions in clinical trials of acupuncture (STRICTA): extending the CONSORT statement. *PLoS Med* 2010;7:e1000261.
8 Gagnier J, Boon H, Rochon P, Moher D, Barnes J, Bombardier C, et al. Reporting randomised, controlled trials of herbal interventions: an elaborated CONSORT statement. *Ann Intern Med* 2006;144:364-7.
9 Chan A, Tetzlaff J, Gøtzsche P, Altman D, Mann H, Berlin J, et al. SPIRIT 2013 explanation and elaboration: guidance for protocols of clinical trials. *BMJ* 2013;346:e7586.
10 Moher D, Schulz K, Simera I, Altman D. Guidance for developers of health research reporting guidelines. *PLoS Med* 2010;7:e1000217.
11 Murphy M, Black N, Lamping D, McKee C, Sanderson C, Askham J, et al. Consensus development methods, and their use in clinical guideline development. *Health Technol Assess* 1998;2:1-88.
12 Von Elm E, Altman D, Egger M, Pocock S, Gøtzsche P, Vandenbroucke J, et al. The Strengthening the Reporting of Observational Studies in Epidemiology (STROBE) statement: guidelines for reporting observational studies. *BMJ* 2007;335:806-8.
13 De Bruin M, Viechtbauer W, Hospers H, Schaalma H, Kok G. Standard care quality determines treatment outcomes in control groups of HAART-adherence intervention studies: implications for the interpretation and comparison of intervention effects. *Health Psychol* 2009;28:668-74.
14 Thorpe K, Zwarenstein M, Oxman AD, Treweek S, Furberg C, Altman D, et al. A pragmatic-explanatory continuum indicator summary (PRECIS): a tool to help trial designers. *J Clin Epidemiol* 2009;62:464-75.
15 Craig P, Dieppe P, Macintyre S, Michie S, Nazareth I, Petticrew M. Developing and evaluating complex interventions: the new Medical Research Council guidance. *BMJ* 2008;337:a1655.
16 McCleary N, Duncan E, Stewart F, Francis J. Active ingredients are reported more often for pharmacologic than non-pharmacologic interventions: an illustrative review of reporting practices in titles and abstracts. *Trials* 2013;14:146.
17 Michie S, West R. Behaviour change theory and evidence: a presentation to Government. *Health Psychol Rev* 2013;7:1-22.
18 Dixon-Woods M, Leslie M, Tarrant C, Bion J. Explaining Matching Michigan: an ethnographic study of a patient safety program. *Implement Sci* 2013;8:70.
19 Dixon-Woods M, Bosk C, Aveling E, Goeschel C, Pronovost P. Explaining Michigan: developing an ex post theory of a quality improvement program. *Milbank Q* 2011;89:167-205.
20 Carroll C, Patterson M, Wood S, Booth A, Rick J, Balain S. A conceptual framework for implementation fidelity. *Implement Sci* 2007;2:40.
21 Bellg AJ, Borrelli B, Resnick B, Hecht J, Minicucci D, Ory M, et al. Enhancing treatment fidelity in health behaviour change studies: best practices and recommendations from the NIH Behaviour Change Consortium. *Health Psychol* 2004;23:443-51.
22 Hardeman W, Michie S, Fanshawe T, Prevost T, McLoughlin K, Kinmonth AL. Fidelity of delivery of a physical activity intervention: predictors and consequences. *Psychol Health* 2008;23:11-24.
23 Spillane V, Byrne M, Byrne M, Leathem C, O'Malley M, Cupples M. Monitoring treatment fidelity in a randomised controlled trial of a complex intervention. *J Adv Nursing* 2007;60:343-52.
24 Hopewell S, Ravaud P, Baron G, Boutron I. Effect of editors' implementation of CONSORT guidelines on the reporting of abstracts in high impact medical journals: interrupted time series analysis. *BMJ* 2012;344:e4178.
25 Hoffmann T, English T, Glasziou P. Reporting of interventions in randomised trials: an audit of journal Instructions to Authors. *Trials* 2014;15:20.
26 Michie S, Fixsen D, Grimshaw JM, Eccles MP. Specifying and reporting complex behaviour change interventions: the need for a scientific method. *Implement Sci* 2009;4:40.
27 Nature. For authors: manuscript formatting guide [Internet]. www.nature.com/nature/authors/gta/index.html#a5.3.
28 Turner L, Shamseer L, Altman DG, Schulz KF, Moher D. Does use of the CONSORT Statement impact the completeness of reporting of randomised controlled trials published in medical journals? A Cochrane review. *Syst Rev* 2012;1:60.
29 Chalmers I, Glasziou P. Avoidable waste in the production and reporting of research evidence. *Lancet* 2009;374:86-9.
30 Glasziou P, Altman D, Bossuyt P, Boutron I, Clarke M, Julious S, et al. Reducing waste from incomplete or unusable reports of biomedical research. *Lancet* 2014;383:267-76.

Accepted: 04 February 2014

Cite this as: *BMJ* 2014;348:g1687

© BMJ Publishing Group Ltd 2014

Summary points

Without a complete published description of interventions, clinicians and patients cannot reliably implement effective interventions

The quality of description of interventions in publications, regardless of type of intervention, is remarkably poor

The Template for Intervention Description and Replication (TIDieR) checklist and guide has been developed to improve the completeness of reporting, and ultimately the replicability, of interventions

TIDieR can be used by authors to structure reports of their interventions, by reviewers and editors to assess completeness of descriptions, and by readers who want to use the information

Tables

Table 1| **Items included in the Template for Intervention Description and Replication (TIDieR) checklist: information to include when describing an intervention. Full version of checklist provides space for authors and reviewers to give location of the information (see appendix 3)**

Item No	Item
**Brief name**	
1	Provide the name or a phrase that describes the intervention
**Why**	
2	Describe any rationale, theory, or goal of the elements essential to the intervention
**What**	
3	Materials: Describe any physical or informational materials used in the intervention, including those provided to participants or used in intervention delivery or in training of intervention providers. Provide information on where the materials can be accessed (such as online appendix, URL)
4	Procedures: Describe each of the procedures, activities, and/or processes used in the intervention, including any enabling or support activities
**Who provided**	
5	For each category of intervention provider (such as psychologist, nursing assistant), describe their expertise, background, and any specific training given
**How**	
6	Describe the modes of delivery (such as face to face or by some other mechanism, such as internet or telephone) of the intervention and whether it was provided individually or in a group
**Where**	
7	Describe the type(s) of location(s) where the intervention occurred, including any necessary infrastructure or relevant features
**When and How Much**	
8	Describe the number of times the intervention was delivered and over what period of time including the number of sessions, their schedule, and their duration, intensity, or dose
**Tailoring**	
9	If the intervention was planned to be personalised, titrated or adapted, then describe what, why, when, and how
**Modifications**	
10*	If the intervention was modified during the course of the study, describe the changes (what, why, when, and how)
**How well**	
11	Planned: If intervention adherence or fidelity was assessed, describe how and by whom, and if any strategies were used to maintain or improve fidelity, describe them
12*	Actual: If intervention adherence or fidelity was assessed, describe the extent to which the intervention was delivered as planned

*If checklist is completed for a protocol, these items are not relevant to protocol and cannot be described until study is complete.

For personal use only: See rights and reprints http://www.bmj.com/permissions Subscribe: http://www.bmj.com/subscribe

Table 2| **List of references for the examples used**

	Citation
1a	Gallagher LTQ, Hill C, Keamy Jr DG, Williams M, Hansen M, Maurer R, et al. Perioperative dexamethasone administration and risk of bleeding following tonsillectomy in children: a randomized controlled trial. *JAMA* 2013;308:1221-6.
1b	Chalder M, Wiles NJ, Campbell J, Hollinghurst SP, Haase AM, Taylor AH, et al. Facilitated physical activity as a treatment for depressed adults: randomised controlled trial. *BMJ* 2012;344:e2758.
1c	Vernooij JWP, Kaasjager HAH, van der Graaf Y, Wierdsma J, Grandjean HMH, Hovens MMC, et al. Internet based vascular risk factor management for patients with clinically manifest vascular disease: randomised controlled trial. *BMJ* 2012;344:e3750.
2a	De Gans JD, van de Beek D. Dexamethasone in adults with bacterial meningitis. *N Engl J Med* 2002;347:1549-56.
2b	Cromheecke ME, Levi M, Colly LP, de Mol BJ, Prins MH, Hutten BA, et al. Oral anticoagulation self-management and management by a specialist anticoagulation clinic: a randomised cross-over comparison. *Lancet* 2000;356:97-102.
2c	Hardeman W, Kinmonth AL, Michie S, Sutton S. Impact of a physical activity intervention program on cognitive predictors of behaviour among adults at risk of type 2 diabetes (ProActive randomised controlled trial). *Int J Behav Nutr Phys Act* 2009;6:16.
2d	Bennell KL, Bowles K, Payne C, Cicuttini F, Williamson E, Forbes A, et al. Lateral wedge insoles for medial knee osteoarthritis: 12 month randomised controlled trial. *BMJ* 2011;342: d2912.
3a	Bieri F, Gray DJ, Williams GM, Raso G, Li Y-S, Yuan L, et al. Health-education package to prevent worm infections in Chinese schoolchildren. *N Engl J Med* 2013;368:1603-12.
3b	Butler CC, Simpson SA, Hood K, Cohen D, Pickles T, Spanou C, et al. Training practitioners to deliver opportunistic multiple behaviour change counselling in primary care: a cluster randomised trial. *BMJ* 2013;346:f1191.
3c	Ekeberg OM, Bautz-holter E, Tveita EK, Juel NG, Kvalheim S. Subacromial ultrasound guided or systemic steroid injection for rotator cuff disease: randomised double blind study. *BMJ* 2009;338:a3112.
4a	Koning G, Andeweg C, Keus F, van Tilburg M, van Laarhoven C, Akkersdijk W. The transrectus sheath preperitoneal mesh repair for inguinal hernia: technique, rationale, and results of the first 50 cases. *Hernia* 2012;16:295-9.
4b	Hijazi R, Taylor D, Richardson J. Effect of topical alkane vapocoolant spray on pain with intravenous cannulation in patients in emergency departments: randomised double blind placebo controlled trial. *BMJ* 2009;338:b215.
4c	Reix P, Aubert F, Werck-Gallois M-C, Toutain A, Mazzocchi C, Moreux N, et al. Exercise with incorporated expiratory manoeuvres was as effective as breathing techniques for airway clearance in children with cystic fibrosis: a randomised crossover trial. *J Physiother* 2012;58:241-7.
4d	Zurovac D, Sudoi RK, Akhwale WS, Ndiritu M, Hamer DH, Rowe AK, et al. The effect of mobile phone text-message reminders on Kenyan health workers' adherence to malaria treatment guidelines: a cluster randomised trial. *Lancet* 2011;378:795-803.
4e	Huang SS, Septimus E, Kleinman K, Moody J, Hickok J, Avery TR, et al. Targeted versus universal decolonisation to prevent ICU infection. *N Engl J Med* 2013;368:2255-65.
5a	Doherty T, Tabana H, Jackson D, Swanevelder S, Fox MP, Thorson A. Effect of home based HIV counselling and testing intervention in rural South Africa: cluster randomised trial. *BMJ* 2013;3481:1-11.
5b	Tiernan J, Hind D, Watson A, Wailoo AJ, Bradburn M, Shephard N, et al. The HubBLe trial: haemorrhoidal artery ligation (HAL) versus rubber band ligation (RBL) for haemorrhoids. *BMC Gastroenterol* 2012;12:153.
5c	Wiles N, Thomas L, Abel A, Ridgway N, Turner N, Campbell J, et al. Cognitive behavioural therapy as an adjunct to pharmacotherapy for primary care based patients with treatment resistant depression: results of the CoBalT randomised controlled trial. *Lancet* 2013;381:375-84.
5d	Kaner E, Bland M, Cassidy P, Coulton S, Dale V, Deluca P, et al. Effectiveness of screening and brief alcohol intervention in primary care (SIPS trial): pragmatic cluster randomised controlled trial. *BMJ* 2013;346:e8501.
6a	Thomas S, Thomas PW, Kersten P, Jones R, Green C, Nock A, et al. A pragmatic parallel arm multi-centre randomised controlled trial to assess the effectiveness and cost-effectiveness of a group-based fatigue management programme (FACETS) for people with multiple sclerosis. *J Neurol Neurosurg Psychiatry* 2013;84:1092-9.
6b	Bojang K, Akor F, Conteh L, Webb E, Bittaye O, Conway DJ, et al. Two strategies for the delivery of IPTc in an area of seasonal malaria transmission in the Gambia: a randomised controlled trial. *PLoS Med* 2011;8:e1000409.
6c	Ybarra M, Bağcı Bosi A., Korchmaros J, Emri S. A text messaging-based smoking cessation program for adult smokers: randomised controlled trial. *J Med Internet Res* 2012;14:e172.
6d	Kessler D, Lewis G, Kaur S, Wiles N, King M, Weich S, et al. Therapist-delivered internet psychotherapy for depression in primary care: a randomised controlled trial. *Lancet* 2009;374:628-34.
6e	Chumbler NR, Quigley P, Li X, Morey M, Rose D, Sanford J, et al. Effects of telerehabilitation on physical function and disability for stroke patients: a randomised, controlled trial. *Stroke* 2012;43:2168-74.
7a	Halterman J, Szilagyi P, Fisher S, Fagnano M, Tremblay P, Conn K, et al. Randomised controlled trial to improve care for urban children with asthma: results of the school-based asthma therapy trial. *Arch Pediatr Adolesc Med* 2013;165:262-8.
7b	Van den Broek NR, White SA, Goodall M, Ntonya C, Kayira E, Kafulafula G, et al. The APPLe study: a randomised, community-based, placebo-controlled trial of azithromycin for the prevention of preterm birth, with meta-analysis. *PLoS Med* 2009;6:e1000191.
7c	Cartwright M, Hirani SP, Rixon L, Beynon M, Doll H, Bower P, et al. Effect of telehealth on quality of life and psychological outcomes over 12 months (Whole Systems Demonstrator telehealth questionnaire study): nested study of patient reported outcomes in a pragmatic, cluster randomised controlled trial. *BMJ* 2013;346:f653.
7d	Lewycka S, Mwansambo C, Rosato M, Kazembe P, Phiri T, Mganga A, et al. Effect of women's groups and volunteer peer counselling on rates of mortality, morbidity, and health behaviours in mothers and children in rural Malawi (MaiMwana): a factorial, cluster-randomised controlled trial. *Lancet* 2013;381:1721-35.

For personal use only: See rights and reprints http://www.bmj.com/permissions Subscribe: http://www.bmj.com/subscribe

Table 2 (continued)

	Citation
7e	Murphy AW, Cupples ME, Smith SM, Byrne M, Byrne MC, Newell J. Effect of tailored practice and patient care plans on secondary prevention of heart disease in general practice: cluster randomised controlled trial. *BMJ* 2009;339:b4220.
8a	Shakur H, Roberts I, Bautista R, Caballero J, Coats T, Dewan Y, et al. Effects of tranexamic acid on death, vascular occlusive events, and blood transfusion in trauma patients with significant haemorrhage (CRASH-2): a randomised, placebo-controlled trial. *Lancet* 2010;376:23-32.
8b	Free C, Knight R, Robertson S, Whittaker R, Edwards P, Zhou W, et al. Smoking cessation support delivered via mobile phone text messaging (txt2stop): a single-blind, randomised trial. *Lancet* 2011;378:49-55.
8c	McDermott MM, Ades P, Guralnik JM, Nelson M, Horn L Van, Garside D, et al. Treadmill exercise and resistance training in patients with peripheral arterial disease with and without intermittent claudication. *JAMA* 2009;301:165-74.
8d	Morrell CJ, Slade P, Warner R, Paley G, Dixon S, Walters SJ, et al. Clinical effectiveness of health visitor training in psychologically informed approaches for depression in postnatal women: pragmatic cluster randomised trial in primary care. *BMJ* 2009;338:a3045.
9a	Stewart S, Carrington MJ, Swemmer CH, Anderson C, Kurstjens NP, Amerena J, et al. Effect of intensive structured care on individual blood pressure targets in primary care: multicentre randomised controlled trial. *BMJ* 2012;345:e7156.
9b	Lee W-J, Wang W, Lee Y-C, Huang M-T, Ser K-H, Chen J-C. Laparoscopic mini-gastric bypass: experience with tailored bypass limb according to body weight. *Obes Surg* 2008;18:294-9.
9c	McDermott MM, Ades P, Guralnik JM, Nelson M, Horn L Van, Garside D, et al. Treadmill exercise and resistance training in patients with peripheral arterial disease with and without intermittent claudication. *JAMA* 2009;301:165-74.
9d	Davidson KW, Rieckmann N, Clemow L, Schwartz JE, Shimbo D. Enhanced depression care for patients with acute coronary syndrome and persistent depressive symptoms. *JAMA Intern Med* 2010;170:600-8.
10a	Kaner E, Bland M, Cassidy P, Coulton S, Dale V, Deluca P, et al. Effectiveness of screening and brief alcohol intervention in primary care (SIPS trial): pragmatic cluster randomised controlled trial. *BMJ* 2013;346:e8501.
10b	Wake M, Lycett K, Clifford SA, Sabin MA, Gunn J, Gibbons K, et al. Shared care obesity management in 3-10 year old children: 12 month outcomes of HopSCOTCH randomised trial. *BMJ* 2013;346:f3092.
11a	Kapiteijn E, Marijnen C, Nagtegaal I, Putter H, Steup W, Wiggers T, et al. Preoperative radiotherapy combined with total mesorectal excision for resectable rectal cancer. *N Engl J Med* 2001;345:638-46.
11b	Morrison AP, French P, Stewart SLK, Birchwood M, Fowler D, Gumley AI, et al. Early detection and intervention evaluation for people at risk of psychosis: multisite randomised controlled trial. *BMJ* 2012;344:e2233.
11c	Thomas KS, Crook AM, Nunn AJ, Foster K, Mason JM, Chalmers JR, et al. Penicillin to prevent recurrent leg cellulitis. *N Engl J Med* 2013;368:1695-703.
11d	Murphy AW, Cupples ME, Smith SM, Byrne M, Leathem C, Byrne MC. The SPHERE Study. Secondary prevention of heart disease in general practice: protocol of a randomised controlled trial of tailored practice and patient care plans with parallel qualitative, economic and policy analyses. *Trials* 2005;16:1-16.
12a	Coombes BK, Bisset L, Brooks P, Khan A, Vincenzio B. Effect of corticosteroid injection, physiotherapy, or both on clinical outcomes in patients with unilateral lateral epicondylalgia: a randomized controlled trial. *JAMA* 2013;309:461-9.
12b	Dodd MJ, Cho MH, Miaskowski C, Krasnoff J, Bank KA. A randomised controlled trial of home-based exercise for cancer-related fatigue in women during and after chemotherapy with or without radiation therapy. *Cancer Nurs* 2010;33:245-57.
12c	Thomas KS, Crook AM, Nunn AJ, Foster K, Mason JM, Chalmers JR, et al. Penicillin to prevent recurrent leg cellulitis. *N Engl J Med* 2013;368:1695-703.
12d	Kessler D, Lewis G, Kaur S, Wiles N, King M, Weich S, et al. Therapist-delivered internet psychotherapy for depression in primary care: a randomised controlled trial. *Lancet* 2009;374:628-34.

For personal use only: See rights and reprints http://www.bmj.com/permissions Subscribe: http://www.bmj.com/subscribe
